# Supplementary material for: The Current Epidemiology of Urinary Incontinence and Urinary Tract Infections After Spinal Cord Injury—A Model Systems Spinal Cord Injury Examination (2016–2021)
Source: J Clin Med. 2025 Feb 21;14(5):1434. doi: 10.3390/jcm14051434 (PMC11899757; doi:10.3390/jcm14051434)
Supplement: Supplementary file 1 [file jcm-14-01434-s001.zip › jcm-3417313-supplementary.pdf]

Table S1. – Factors Associated with Frequent Urinary Incontinence (Weekly or More) in the Model System Spinal Cord Injury Cohort (2016-2021) (Year 1 & Year 5 follow-up cohorts combined). .

		Univariate		Multivariate	
		OR (95%CI)	p-value	OR (95% CI)	p-value
	Age at Injury (years)	1.00 (0.99-1.01)	0.448	1.01 (1.01-1.02)	<0.001
Sex	Male	Ref	-	Ref	-
	Female	1.39 (1.18-1.63)	<0.001	1.68 (1.41-2.00)	<0.001
Race/Ethnicity	White	Ref	-	Ref	-
	Black	0.97 (0.82-1.14)	0.696	0.95 (0.80-1.14)	0.601
	American Indian, Alaska Native	1.00 (0.48-2.10)	0.991	0.93 (0.41-2.10)	0.865
	Asian, Pacific Islander	0.95 (0.60-1.49)	0.807	1.06 (0.65-1.73)	0.807
	Other, Multiracial	1.10 (0.78-1.55)	0.575	1.08 (0.75-1.56)	0.667
Bladder Management at Follow-up	Volitional Void	Ref	-	Ref	-
	Condom Cath/Diapers	2.54 (1.87-3.46)	<0.001	2.31 (1.66-3.20)	<0.001
	Indwelling Catheter	0.80 (0.63-1.01)	0.062	0.60 (0.44-0.82)	0.001
	CIC	3.02 (2.55-3.59)	<0.001	2.72 (2.13-3.50)	<0.001

	Conduit	0.76 (0.96-6.01)	0.794	0.55 (0.07-4.66)	0.589
	Other	3.56 (1.75-7.26)	<0.001	3.41 (1.63-7.16)	0.001
Number of UTI in the last year treated with antibiotics	No UTI	Ref	-	Ref	-
	1 or 2 UTI's	1.48 (1.24-1.75)	<0.001	1.23 (1.01-1.49)	0.031
	3 or 4 UTIs	2.04 (1.68-2.48)	<0.001	1.71 (1.37-2.14)	<0.001
	5 or more UTI's	1.85 (1.48-2.32)	<0.001	1.74 (1.35-2.25)	<0.001
Year of Follow-up	1 Year	Ref	-	Ref	-
	5 Year	0.91 (0.79-1.04)	0.169	0.97 (0.84-1.13)	0.716
AIS Class	A	Ref	-	Ref	-
	B	0.62 (0.49-0.78)	<0.001	0.69 (0.54-0.89)	0.005
	C	0.57 (0.45-0.71)	<0.001	0.63 (0.50-0.82)	<0.001
	D	0.52 (0.44-0.62)	<0.001	0.74 (0.56-0.97)	0.029
Injury Level	Cervical	Ref	-	Ref	-
	Thoracic	1.91 (1.65-2.22)	<0.001	1.28 (1.05-1.55)	0.011
	Lumbosacral	1.40 (1.07-1.85)	0.014	1.28 (0.96-1.73)	0.094

Table S2. – Factors Associated with Recurrent Urinary Tract Infections (3 or More UTI in the Past Year) in the Model System Spinal Cord Injury Cohort (2016-2021) (Year 1 & Year 5 follow-up cohorts combined). .

		Univariate		Multivariate	
		OR (95%CI)	p-value	OR (95% CI)	p-value
	Age at Injury (years)	0.99 (0.98-0.99)	<0.001	0.99 (0.98-0.99)	0.024
Sex	Male	Ref	-	Ref	-
	Female	1.31 (1.13-1.52)	<0.001	1.47 (1.23-1.75)	<0.001
Race/Ethnicity	White	Ref	-	Ref	-
	Black	0.90 (0.77-1.05)	0.181	0.95 (0.79-1.13)	0.549
	American Indian, Alaska Native	1.53 (0.82-2.86)	0.183	1.34 (0.63-2.83)	0.443
	Asian, Pacific Islander	0.99 (0.67-1.50)	0.992	1.10 (0.68-1.79)	0.676
	Other, Multiracial	0.89 (0.64-1.24)	0.482	0.73 (0.50-1.04)	0.087
Bladder Management at Follow-up	Volitional Void	Ref	-	Ref	-
	Condom Cath/Diapers	3.10 (2.08-4.61)	<0.001	2.60 (1.72-3.95)	<0.001
	Indwelling Catheter	13.03 (10.26-16.55)	<0.001	10.85 (8.03-14.64)	<0.001
	CIC	7.95 (6.32-10.00)	<0.001	5.68 (4.26-7.57)	<0.001
	Conduit	4.35 (0.91-20.78)	0.065	2.27 (0.24-21.21)	0.473

	Other	5.16 (2.28-11.66)	<0.001	4.20 (1.80-9.78)	0.001
Urinary Incontinence	None	Ref	-	Ref	-
	Daily incontinence	2.11 (1.72-2.58)	<0.001	1.90 (1.53-2.38)	<0.001
	Weekly incontinence	1.77 (1.43-2.18)	<0.001	1.48 (1.18-1.86)	0.001
	Monthly incontinence	1.81 (1.51-2.18)	<0.001	1.37 (1.12-1.67)	0.002
Year of Follow-up	1 Year	Ref	-	Ref	-
	5 Year	0.84 (0.72-0.95)	0.007	0.75 (0.65-0.87)	<0.001
AIS Class	A	Ref	-	Ref	-
	B	0.76 (0.62-0.92)	0.005	0.85 (0.68-1.06)	0.141
	C	0.58 (0.48-0.70)	<0.001	0.87 (0.69-1.09)	0.238
	D	0.18 (0.15-0.22)	<0.001	0.73 (0.55-0.96)	0.025
Injury Level	Cervical	Ref	-	Ref	-
	Thoracic	1.28 (1.12-1.47)	<0.001	1.10 (0.92-1.33)	0.291
	Lumbosacral	0.90 (0.63-1.29)	0.567	0.87 (0.62-1.15)	0.317
